# A medically grounded LLM agent–based tool to detect patient safety events in medical records

**DOI:** 10.1371/journal.pdig.0001174

**Published:** 2026-09-17

**Authors:** Diego Trujillo, Dulin Wang, Nathan Bahr, Tina Yi-Jin Hsieh, Byeongyeon Cho, Garth Meckler, Matthew Hansen, Carl Eriksson, Kyu Seo Kim, Steven Bedrick, Xiaoqian Jiang, Jeanne-Marie Guise

**Affiliations:** 1 Center for Learning Health Care Delivery, Beth Israel Deaconess Medical Center, Boston, Massachusetts, United States of America; 2 Department of Obstetrics Gynecology and Reproductive Medicine, Beth Israel Deaconess Medical Center and Harvard Medical School, Boston, Massachusetts, United States of America; 3 McWilliams School of Biomedical Informatics, UTHealth, Houston, Texas, United States of America; 4 Department of Emergency Medicine, Oregon Health and Science University, Portland, Oregon, United States of America; 5 Department of Biomedical Informatics, Harvard Medical School, Boston, Massachusetts, United States of America; 6 Department of Pediatrics and Emergency Medicine, University of British Columbia, Vancouver, Canada; 7 Department of Pediatrics, Oregon Health and Science University, Portland, Oregon, United States of America; 8 Department of Medical Informatics and Clinical Epidemiology, Oregon Health and Science University, Portland, Oregon, United States of America; Chinese PLA General Hospital, CHINA

## Abstract

Large language models (LLMs) have shown incredible promise in medicine. While LLMs may be particularly useful in areas requiring extensive review of clinical records, their use remains limited due to their tendency to hallucinate and fabricate information. Hallucination issues, as well as their consequences, are exacerbated in low–probability, high–stakes scenarios such as rare adverse safety events or medical errors. We present SAFE–AI (Structured and Automated Framework for Explainable AI), a novel method for clinical decision making that combines the strengths of clinical expert knowledge with LLMs in an ontology–driven model that minimizes hallucinations using strict rules. We test this method to identify medication errors in medical charts. We collected a sample of 18,402 lines of clinical information from 300 EMS clinical charts that were independently dually reviewed by two expert physicians for epinephrine adverse safety events (ASEs), with 96% inter-rater agreement. We tested SAFE–AI against these labels, achieving similar performance to human experts in detecting epinephrine overdoses with 97.9% accuracy, and 91.6% accuracy in identifying delays in epinephrine administration, greatly outperforming baseline LLMs models. Notably, some disagreements between clinicians and the model were found to be justifiable differences in judgment rather than errors. SAFE-AI presents a novel approach for clinical AI applications that addresses two key limitations of current machine learning methods: 1) over-reliance on probabilistic pattern recognition instead of established medical knowledge, and 2) perpetuation of biases present in training data. This framework is easily adaptable to a range of clinical applications, paving the way for provable and trustworthy AI in medicine.

## Author summary

LLMs have shown promise in analyzing clinical records but their use is limited due to their tendency to hallucinate and fabricate information. Misinformation could threaten patient safety and jeopardize trust. We developed SAFE–AI (Structured and Automated Framework for Explainable AI), which combines knowledge from clinical experts with LLM inference to detect adverse safety events (ASEs) with minimal errors. We tested our method in identifying medication errors in medical charts and compared results to reviews by expert physicians. Our method detected epinephrine delays and overdoses with a high level of accuracy. SAFE-AI presents a novel approach for clinical AI applications that overcomes reliance on pattern recognition instead of medical knowledge biases present in training data.

## Introduction

It is estimated that over 250,000 adverse safety events (ASEs) occur annually in the United States among patients receiving medical care, resulting in more than 100,000 patient deaths [1–3]. ASEs, such as medication, diagnostic, or procedural errors; miscommunications; and healthcare associated infections, pose significant risks to patient safety in healthcare settings. The current standard for detecting ASEs is for expert clinicians to manually review clinical records [4,5]. Manual chart reviews are labor-intensive, slow, and costly. Consequently, ASEs are undetected and under-reported, with over 95% of documented ASEs in medical records not being included in the official records, which hampers quality improvement and patient safety efforts [6].

Recently, artificial intelligence (AI) and particularly large language models (LLMs), have shown promise for augment- ing the abilities of healthcare professionals to analyze complex medical data. However, these models have limitations such as the propensity for LLMs to “hallucinate”, that is, to produce fictional or fabricated information that can be misleading and dangerous in clinical settings [7–9]. Recent studies have shown that their performance is highly conditional on the probability of the correct outcome [10]. As ASEs are relatively uncommon events, LLMs alone are not likely to correctly identify them. Even more troubling are some recent analyses of LLM robustness, showing the results of LLM requests are greatly dependent on the specific phrasing used [11].

LLM hallucination is most evident when the model is asked to perform reasoning-based tasks, but is greatly mitigated when limited to extracting information. This is demonstrated by the success of techniques such as retrieval-augmented generation (RAG) [12], where the prompt is augmented with additional context from relevant databases before query- ing the LLM. RAG improves LLM performance and reduces hallucinations by up to 26% [13]. By including context, and potentially the solution, in the prompt, the model acts as an information retrieval system. However, while RAG is successful in improving the results of LLMs, it does not offer any true protection against hallucinations, and still behaves as a black box, lacking explainability and verifiability.

To address these challenges, we developed SAFE–AI (Structured and Automated Framework for Explainable AI), an automated decision-making framework that merges state-of-the-art AI models with structured clinical knowledge. By combining the capabilities of LLMs with dynamic prompting and automatic decision–making based on deterministic logic, SAFE–AI offers fast and reliable identification of ASEs from both free-text clinical notes and structured data sources. Our approach seeks to bridge the gap between clinician knowledge and fast, automatic ASE detection. Specifically, we test the ability of SAFE-AI in reviewing EMS charts for medication errors, specifically potentially lethal overdoses or delays in administering Epinephrine in pediatric out-of-hospital cardiac arrest (OHCA) care [14]. We test this approach on epinephrine dosing and timing (two serious ASEs in OHCA), and compare against baseline LLMs and human reviewers.

## Methods

### Overview

**Fig 1:**
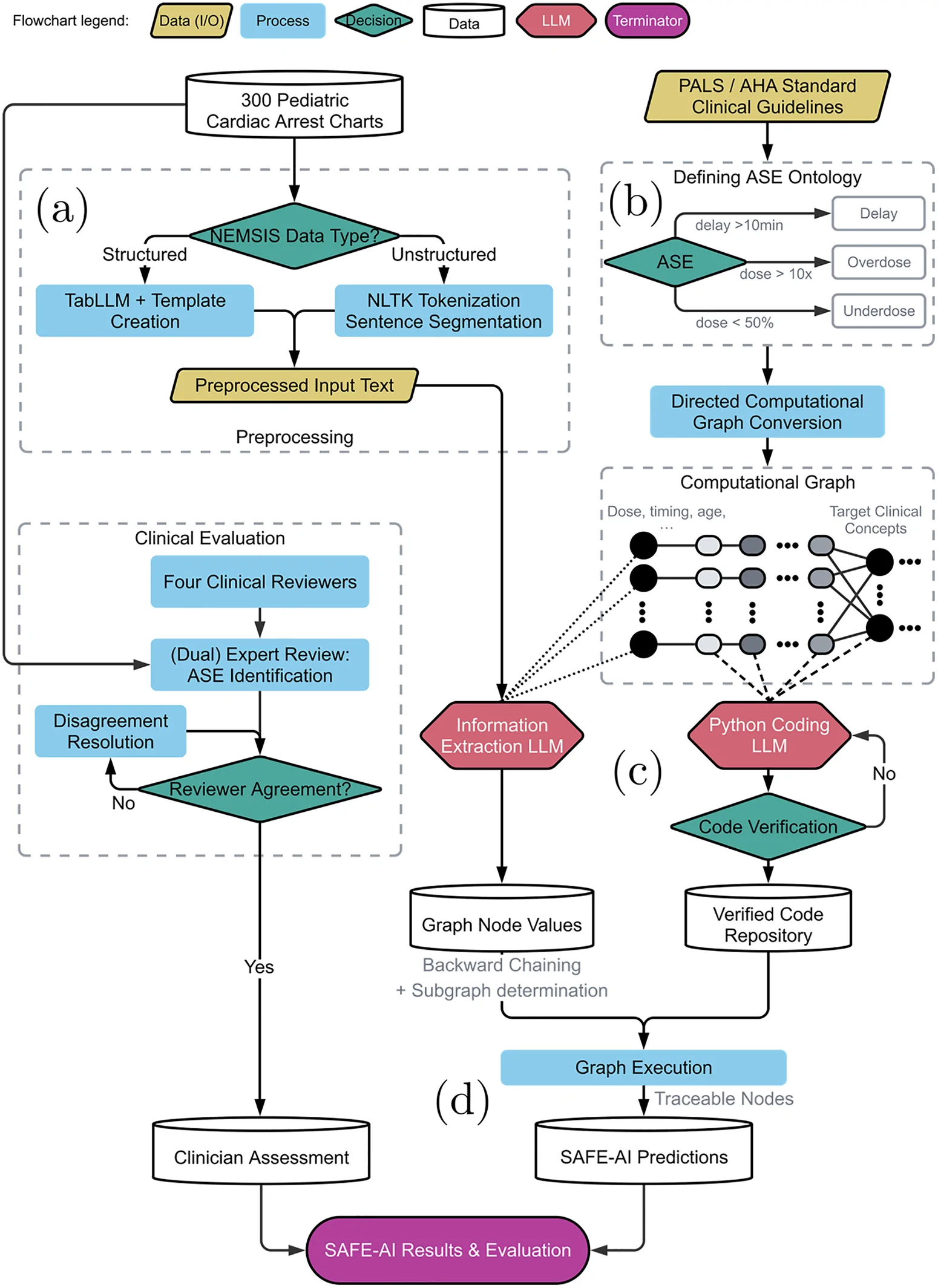
SAFE–AI process diagram. **a)** An emergency medical services (EMS) chart containing both structured and unstructured information is taken as input; **b)** an ontology of ASE–related concepts is defined, also defining relationships between concepts; **c)** inputs to the ontology are extracted from the text and a coding LLM translates the guidelines into executable code for the intermediate nodes in the ontology; **d)** the complete program makes a final determination by executing the graph with the extracted inputs.^1^Created in BioRender. Hsieh, T. (2025) https://BioRender.com/r57y548.

SAFE–AI is structured in four major steps (Fig 1). Firstly, an ontology of epinephrine errors is defined by clinician experts using established clinical guidelines, and this ontology is converted into a directed graph. Secondly, an LLM is used to retrieve initial nodes in the graph - such as patient age and weight from an unstructured patient chart. Thirdly, a coding LLM translates the guidelines defined in the ontology into executable code. Lastly, a final prediction is made by executing the computational graph of all nodes from the initial inputs retrieved by the LLM to the final output node. SAFE–AI is similar to RAG approaches as the LLM output is conditioned on contextual information, however, it separates this extraction step from decision-making. Thus, the only source of hallucination stems from the initial information extraction - a far simpler task for LLMs to perform accurately.

### Data collection

**Fig 2:**
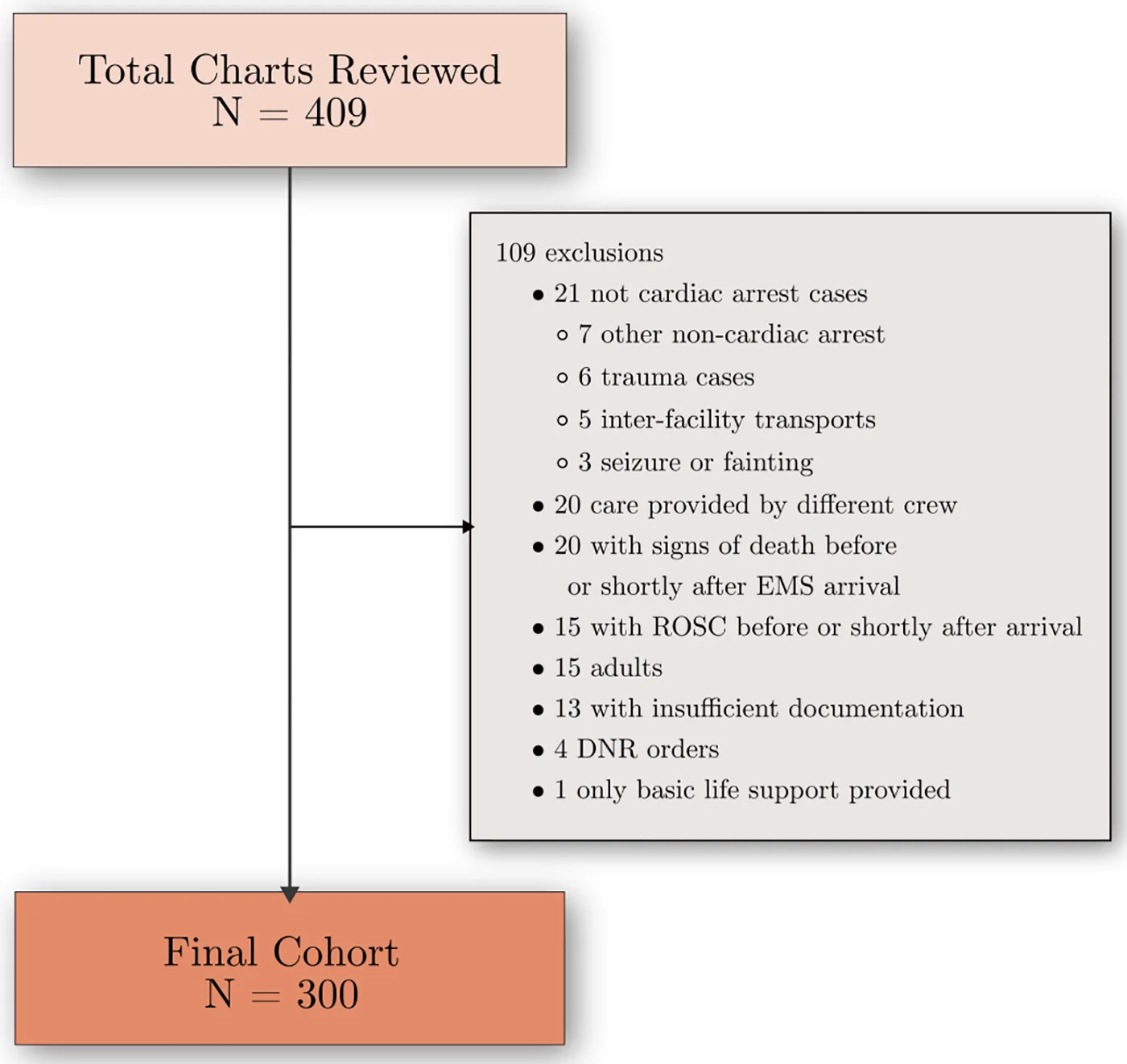
Excluded and reviewed charts.

We acquired a sample of 5,089 pediatric OHCA patient care charts from 2014 to 2019 from a large nationwide provider of EMS transports using the National Emergency Medical Services Information System (NEMSIS) standard [15]. The study was approved by Beth Israel Deaconess Medical Center’s Institutional Review Board (IRB#2022P000948). We randomly selected 409 charts to build a gold standard and test our system’s performance. Inclusion/exclusion criteria are shown in Fig 2. The final sample contained 300 charts and 18,402 lines of clinical information.

Clinical experts in pediatric Emergency Medicine and Critical Care independently verified inclusion status and de- termined presence of ASEs. Following a process described in previous studies [14,16,17], board-certified clinical experts used a validated PEDS tool to independently review medical records for ASEs. Reviewers underwent rigorous training and were required to have at least 80% agreement before they were allowed to independently review. Every chart was randomly assigned to two independent reviewers and disagreements were resolved by complete consensus.

Four clinical experts in pediatric Emergency Medicine and Critical Care verified inclusion status and determined presence of ASEs. Three of our four reviewers had served as the gold standard for severe ASE assessment in our prior work [16]; the fourth underwent training prior to review to ensure they had at least 80randomly assigned to two independent reviewers and disagreements were resolved by complete consensus.

We used Gwet’s AC1 to measure inter-rater agreement for epinephrine ASEs [18]. We have used this measure in previous studies [19] to overcome the “kappa paradox” [20], wherein the low prevalence of events, such as severe ASEs, could result in low scores despite high agreement among raters [18,21,22].

### Data preprocessing

Charts in the National Emergency Medical Services Information System (NEMSIS) standard contain unstructured narratives and structured fields [15]. We used manually-crafted templates, such as “the patient weighs X kg”, to generate sentences from structured fields, following the strategy used by TabLLM [23]. Unstructured narratives were tokenized into sentences with Python’s Natural Language Toolkit [24]. The tokenized and templated sentences were combined into a single document and used as input for the LLM to perform information extraction.

### Ontology of epinephrine errors

To demonstrate the capabilities of the SAFE–AI framework, we created an ontology to formalize epinephrine ASEs. In previous work [16,17] we created a classification system that defined epinephrine ASE as failure to administer when indicated, a delay to administer epinephrine within 10 minutes of arrival, and administering at least a 10x overdose or 50% underdose. In this work, we focused on the latter three. To build an ontology, we expanded these definitions with explicit criteria for prerequisites, and constraints.

Epinephrine is indicated in all pediatric pulseless cardiac arrest according to Pediatric Advanced Life Support (PALS) guidelines published by the American Heart Association (AHA) [25]. Using expert consensus and rules developed in prior studies, we developed a specific ontology including specific time thresholds and erroneous doses that represent ASEs with the potential to negatively impact patient outcomes. We defined thresholds for severe ASE, mild ASE, and no ASE (Table 1), and included the generated ontologies for overdose, underdose, and delay in administration (Fig 1).

**Table 1:** Criteria for severe epinephrine adverse safety events (ASEs).

ASE Type	Criteria for Mild ASE	Criteria for Severe ASE
Delay in epinephrine administration	First dose of epinephrine given *≥* 5 minutes after (1) EMS arrival and (2) beginning of cardiac arrest	First dose of epinephrine given *≥* 10 minutes after (1) EMS arrival and (2) beginning of cardiac arrest
Epinephrine overdose	Administration of *≥* 2 times the recommended epinephrine dose (0.01 mg/kg)	Administration of *≥* 10 times the recommended epinephrine dose (0.01 mg/kg)
Epinephrine underdose	Administration of *≤* 80% of the recommended epinephrine dose (0.01 mg/kg)	Administration of *≤* 50% of the recommended epinephrine dose (0.01 mg/kg)

### LLMs as zero-shot information extractors

LLMs, such as GPT–5, have demonstrated strong performance in extracting structured information from unstructured text. In this work, we leverage this capability while removing the need of LLM reasoning for clinical decision-making. Our design is motivated by the observation that extracting explicitly stated information (e.g., patient age, weight, medication doses, and timestamps) is a significantly easier task than performing multi-step clinical reasoning, which often requires strict adherence to thresholds defined in guidelines. In preliminary experiments, we found that LLM reasoning frequently led to inconsistencies and errors. More details in the Results section.

Therefore, we restrict the role of the LLM to zero-shot information extraction. Specifically, we use the model to identify and extract relevant entities defined by the epinephrine error ontology (Table 1) from clinical narratives. The extracted variables are then passed to a deterministic, rule-based system for ASE determination. To accomplish this, we combine the ontology with a template-based prompting system that dynamically generates queries and structures the outputs into JSON format. This design allows us to leverage the strengths of LLMs in parsing unstructured text while ensuring that clinical decisions are controlled by explicit, verifiable logic.

### Ontologies as computational graphs

Once the relevant inputs have been extracted from the source text with the LLM, a decision can be made with verifiable 93 logic. Using template-based prompting, a coding model (GPT 5.4 mini) is used during development to translate clinical guidelines into Python functions. Specifically, we generated the code offline and ran it in a sandboxed environment. Then, domain experts manually reviewed and validated the generated code to ensure fidelity to clinical guidelines, in addition to basic compilation errors or malicious code, and applied wrapper functions to handle errors and rounding of floating–point numbers. These verified functions are then fixed and reused across all experiments. This design promotes deterministic ASE detection.

When trying to determine an ASE, SAFE–AI selects the relevant output node in the graph and determines the required subgraph of relevant nodes with backward chaining instead of evaluating the full graph. Specifically, starting from the target node (i.e., ASE determination), we recursively traverse its dependencies in the ontology graph to identify all required ancestor nodes. This results in a minimal subgraph containing only the variables necessary for computing the target. Then, after initial nodes in the graph receive their values from the LLM extraction and are coerced to expected data types, the computational graph is executed from the input nodes to the output node. In case of missing inputs or any errors during execution, the program propagates the error and allows tracing it back its origin.

By treating the ontology as a computational graph where nodes represent concepts such as patient age, administered doses, or dosing ASEs, we ensure traceability of the decision-making. All the intermediate and output nodes have explicit computations that can be verified for accuracy, and only need to be defined once.

## Results

### Clinician annotation and inter-rater agreement

A final sample of 300 pediatric emergency medical charts was reviewed by clinician experts. Gwet’s AC1 showed excellent inter–rater agreement among clinical reviewers for evaluating epinephrine ASEs, with values of 0.85 for delays of *≥* 10 minutes to administer epinephrine and 0.96 for incorrect epinephrine doses.

**Fig 3:**
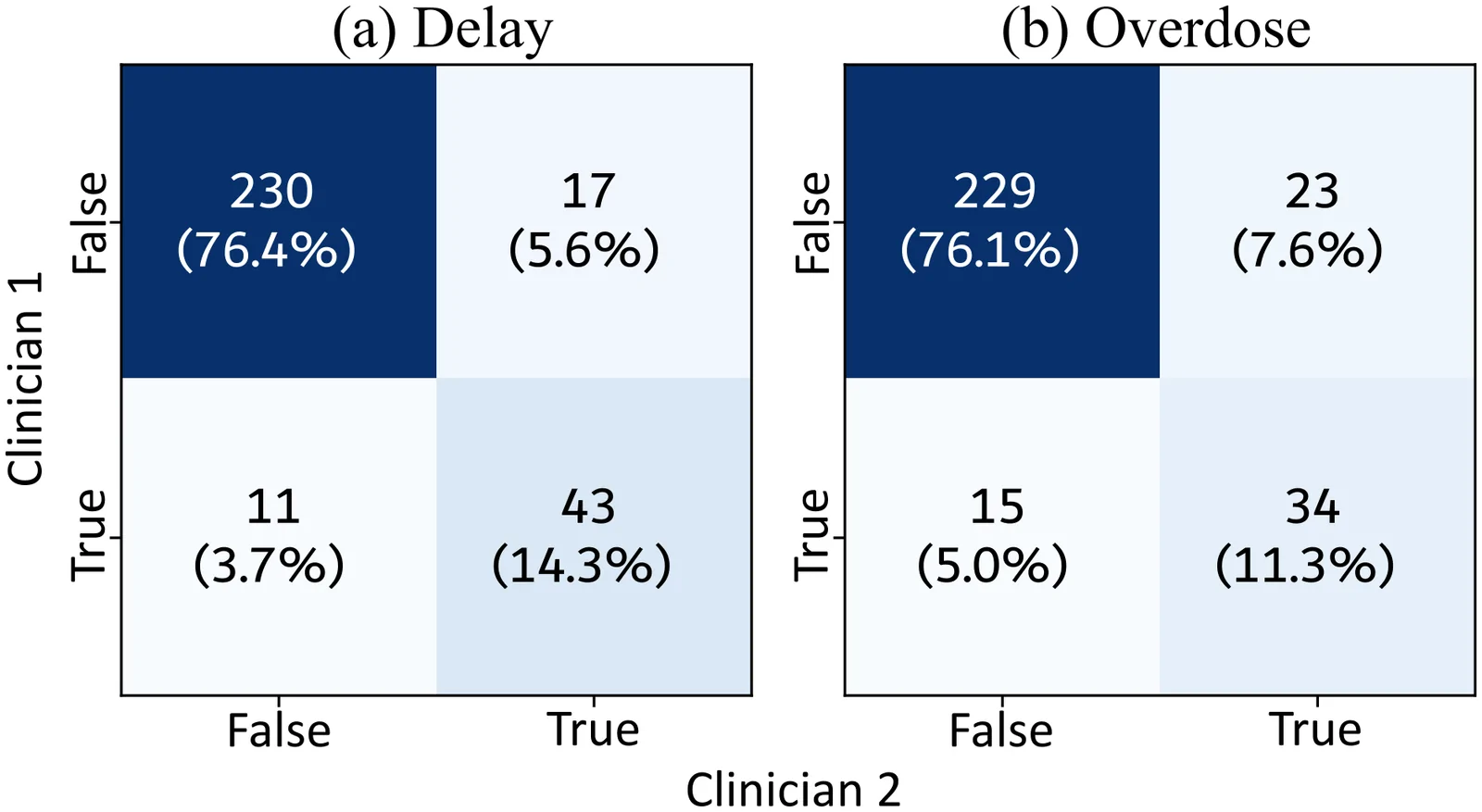
Experts percent agreement for epinephrine ASEs.

We analyzed annotator behavior via pairwise confusion matrices and percent agreement across error categories (Fig 3). We observe that the agreement varied by type of ASE and, in general, reflected clinical ambiguity rather than random disagreement. For instance, in the case of epinephrine indication errors, clinicians agreed in most cases, with disagreements primarily occurring in “scoop-and-run” scenarios where rapid transport was prioritized over on-scene medication administration. These cases frequently fell close to the pre-specified 10-minute threshold, leading to disagreement regarding whether the event constituted a failure to administer epinephrine versus a justified delay. Dosing error annotations demonstrated high agreement, consistent with the high AC1 value reported above.

### Ablated baselines

In order to evaluate the importance of ontological relationships and decision making logic code as opposed to raw next–token prediction, we implemented two ablated baselines. These baselines remove the computational layer of decision making functions, relying on the LLM’s direct interpretation of the medical case and context to make a decision.

**LLM-only baseline.** In this approach, all relevant information (clinical guidelines, task definition, and input case) is provided in a single prompt, and the LLM is asked to directly determine whether an ASE occurred or not. This corresponds to a single-step inference setting, where the model must internally organize the reasoning process without explicit intermediate structure.

**LLM with ontology baseline.** In contrast, the second baseline incorporates a structured decomposition by following the ontology graph and predicting each entity in topological order, conditioning on previously inferred variables. To accomplish this, the baseline uses a prompt template that includes the medical case text, entity metadata (name, description, type), allowed value constraints, and previously computed dependent entity values as context. This is conceptually similar to retrieval–augmented generation, in that the model token generation is conditioned on helpful context, which can be described as a context-augmented generation with endogenous variables instead of retrieval from external documents.

**Fig 4:**
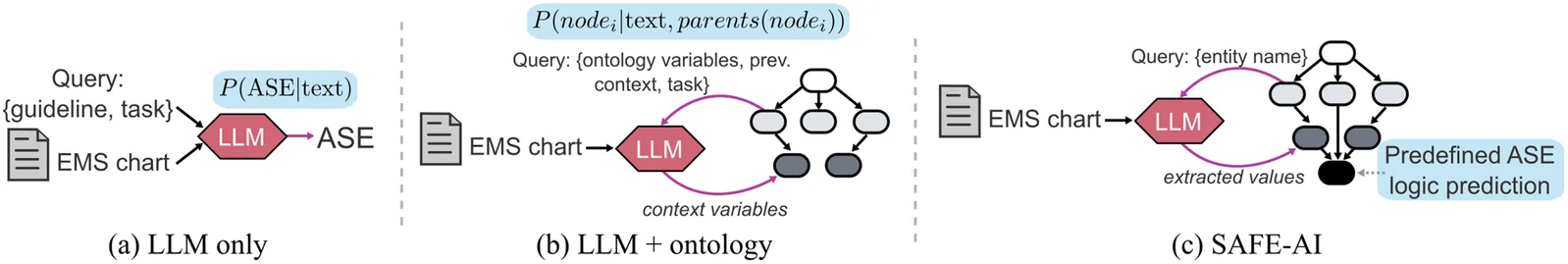
Overview of the compared baselines. **(a)** The LLM-only baseline performs monolithic inference, directly predicting ASE outcomes from the raw clinical text without explicit structure. **(b)** The LLM + Ontology baseline introduces structured where the LLM predicts intermediate variables following the ontology graph and conditions on previously inferred values. **(c)** In contrast, our SAFE-AI approach only resorts to LLM for entity extraction, and ASE determination is performed via deterministic, rule-based execution over the ontology, removing LLM reasoning at inference time.

**SAFE-AI.** In comparison, our final framework resorts to the LLM as an entity extractor rather than as a reasoner for ASE determination. While it operates over the same ontology graph, SAFE-AI replaces probabilistic node inference with deterministic, rule-based execution. Specifically, at each node, including the final ASE determination, is com- puted via predefined functions that enforce clinical rules and thresholds, and thus knowledge is explicitly encoded, which is critical for clinical domains (Fig 4).

### Epinephrine ASE detection

We applied SAFE–AI with the GPT 5.4 mini model and evaluated our pipeline using the 300 validated and reviewed 144 charts from Fig 2. To evaluate the contribution of ontological structures and decision-making logic in SAFE–AI, we also evaluated and compared performance with the ablated baselines defined in the previous subsection, quantitative results are summarized in Table 2.

**Table 2:** Model results against baselines and ablations.

Metric	LLM-Only Baseline LLM + Ontology SAFE–AI		
*Overdose*			
Accuracy	0.80	0.72	**0.85**
Precision	0.72	0.67	**0.85**
Recall	**0.90**	0.76	0.79
F1-Score	0.80	0.71	**0.82**
*Delay*			
Accuracy	0.93	0.92	**0.94**
Precision	0.66	**0.75**	0.69
Recall	**0.98**	0.67	**0.98**
F1-Score	0.78	0.70	**0.81**

### LLM-only predictions are highly sensitive but prone to overcalling ASEs

The LLM-only baseline consistently favored sensitivity over specificity, identifying nearly all clinically relevant ASEs at the expense of additional false positive predictions. Qualitative review revealed that these errors frequently arose from local reasoning over isolated chart fragments without enforcing global consistency across the clinical timeline.

For example, the model occasionally inferred delayed epinephrine administration from sparse or ambiguously docu- mented timestamps despite evidence elsewhere in the chart indicating timely dosing. Similarly, overdose predictions were sometimes triggered by incomplete aggregation of repeated medication entries or by interpreting narrative men- tions as distinct administered doses.

These behaviors suggest that, while modern LLMs are highly effective at retrieving clinically relevant information from free-text narratives, unconstrained decision-making remains vulnerable to context fragmentation and overinter- pretation when performing final ASE determination directly from text.

### Ontological prompting improves consistency but introduces conservative behavior

Incorporating ontological dependencies reduced several of the inconsistency patterns observed in the LLM-only base- line, particularly for overdose detection, where structured dependencies between patient weight, age, dose thresholds, and medication events improved logical coherence. Compared with the unconstrained baseline, the ontology-guided approach produced fewer spurious overdose predictions and more consistent internal reasoning.

However, the same constraints also introduced a more conservative prediction behavior. Error analysis showed that when intermediate ontology nodes were extracted incorrectly or left unresolved, downstream ASE determination fre- quently defaulted to negative predictions. This effect was especially apparent in delay detection, where small incon- sistencies in timestamp extraction propagated through the dependency graph and prevented otherwise plausible ASE identification. These findings suggest that ontology-guided prompting improves structural consistency, but remains fundamentally limited by the probabilistic nature of LLM-based reasoning at the final decision stage.

### SAFE–AI improves decision consistency through deterministic reasoning

SAFE–AI achieved the strongest overall balance between sensitivity and specificity by separating information extrac- tion from clinical decision-making. Unlike both baselines, SAFE–AI used the LLM exclusively to retrieve structured entities from the chart, while ASE determination itself was performed through explicit deterministic rule execution derived from clinical guidelines. This design substantially reduced hallucinated reasoning paths and improved consis- tency across complex temporal and dosage-related scenarios.

Qualitative review demonstrated that SAFE–AI particularly improved cases involving multi-step temporal reason- ing and cumulative dose interpretation. For instance, the framework correctly handled charts containing multiple epinephrine administrations interleaved with ROSC events, where the LLM-only baseline often failed to maintain a coherent temporal reference frame. Similarly, deterministic dose calculations prevented several overdose hallucina- tions caused by duplicate narrative mentions or ambiguous documentation structure.

### Statistical analysis

Paired McNemar analyses further supported these findings (Table 3). The test focuses on discordant predictions between models, where *b* denotes cases correctly identified by SAFE–AI but missed by the comparator, and *c* denotes cases correctly identified by the comparator but missed by SAFE–AI. Across both delay and overdose detection, SAFE–AI consistently yielded a larger number of uniquely correct predictions relative to the LLM-only baseline (e.g., *b* = 34*, c* = 0 for delay; *b* = 40*, c* = 0 for overdose), indicating a systematic advantage in cases where the models disagree. In contrast, comparisons against the LLM + Ontology baseline showed no discordant pairs (*b* = *c* = 0), suggesting that both approaches produced identical correctness patterns on this evaluation set.

**Table 3:** Paired McNemar comparison between SAFE–AI and baselines.

Comparison	ASE	b	c	Interpretation
SAFE–AI vs LLM-only	Delay	34	0	SAFE–AI favored
SAFE–AI vs LLM-only	Overdose	40	0	SAFE–AI favored
SAFE–AI vs LLM+Ontology	Delay	0	0	Identical outcomes
SAFE–AI vs LLM+Ontology	Overdose	0	0	Identical outcomes

### Error analysis

Error analysis revealed that the remaining SAFE–AI failures were dominated by ambiguity and inconsistency inher- ent to real-world clinical documentation. The largest source of disagreement arose from inter-reviewer variability, highlighting that a substantial subset of charts were themselves difficult to adjudicate even for expert reviewers. This finding suggests that some apparent model “errors” reflect uncertainty in the underlying clinical labels rather than algorithmic failure.

Among true model-related errors, most failures originated during information extraction rather than during ontology execution. In particular, formatting inconsistencies, incomplete timestamps, and conflicting documentation between structured and narrative chart components occasionally propagated incorrect or missing entities into the reasoning pipeline. Fewer failures were attributable to explicit logical reasoning errors, indicating that once the relevant clinical entities were correctly extracted, the symbolic execution framework remained largely stable and internally consistent (Table 4).

**Table 4:** Taxonomy of failure modes in ASE detection.

Failure mode	Frequency	Type/effect	Interpretation
Reviewer disagreement	28/300 (9.3%)	Model correct?	Inter-reviewer variability; resolved via consensus
Extraction failures	22/300 (7.3%)	FN	Formatting/ parsing errors or empty variable values
Dose hallucination	6/300 (2%)	FP	Model invents or mis-aggregates medication doses
Logical errors	6/300 (2%)	FN	Correct extracted entities, incorrect decision logic
Wrong delay reference	5/300 (1.6%)	FP	Incorrect temporal anchor (arrival vs arrest/pre-arrival care)
Late-dose strictness	5/300 (1.6%)	FP	Clinically acceptable delay due to many timely prior doses
Narrative-structure mismatch	5/300 (1.6%)	FP	Conflicting information between free text and structure timestamps
Data inconsistency	1/300 (0.3%)	Excluded	Irreconcilable chart documentation errors

## Discussion

In this study, we proposed SAFE–AI, a framework that combines flexible language understanding with explicit sym- bolic reasoning grounded in clinical guidelines. We demonstrated the capabilities of SAFE–AI in detecting overdoses and delays in medication administration in clinical charts, and found that SAFE–AI shows strong overall performance and consistently competitive or superior F1-scores relative to the evaluated baselines. While LLM-only approaches often achieved high recall, they were more prone to false-positive predictions and inconsistent reasoning behavior, particularly in complex cases. In contrast, SAFE–AI, which combines probabilistic information extraction with the deterministic execution of clinical rules, resulted in more stable and interpretable behavior regardless of the underlying LLM model used and the format of clinical notes.

In spite of the advancements made by AI, manual chart reviews by clinicians remain the gold standard in ASE detec- tion, particularly in out–of–hospital settings [5]. Focused reviews are the most accurate method, but are inadequate for timely intervention and can only be performed retrospectively. Because manual review requires substantial time and clinical expertisechart are time and resource intensive, only a fraction of charts can realistically be reviewed, despite medical errors remaining a major source of morbidity and mortality in the United States [3,26–33].

Our results demonstrate the potential of SAFE–AI as a fast and scalable framework for ASE detection that could assist healthcare institutions to identify safety events shortly after chart completion, enabling earlier review and po- tentially timely corrective action. Moreover, SAFE–AI substantially reduces review time compared to expert manual abstraction. On average, specialized clinician reviewers required more than 10 minutes to review a two-page chart, whereas SAFE–AI completed the same task in under one minute. These findings suggest that symbolic clinical AI sys- tems may help scale patient safety surveillance and quality improvement efforts while maintaining clinically auditable decision-making.

SAFE–AI is not without limitations. Although this study used a nationwide pediatric OHCA dataset containing various EMS documentation styles and clinical scenarios, the evaluation was limited to 300 charts and focused specifically on epinephrine adverse safety events. Additional validation across broader patient populations and institutions will be required to fully characterize the generalizability of the framework. Furthermore, SAFE–AI currently depends on ontologies which require substantial domain expertise and manual development effort. While this explicit knowledge representation improves interpretability, extending the framework to additional ASE domains will require the creation and validation of new ontologies. Future work will focus on expanding the framework to additional clinical domains, evaluating performance on larger multi-center datasets and incorporating more advanced verification strategies

Although this proof-of-concept study was limited to a low-frequency event (epinephrine administration in pediatric OHCA), this high value intervention [34] is a common medical error in the out-of-hospital setting [35] and its detection continues to rely on manual chart review and human calculation [36]. We believe that clinically grounded frameworks such as SAFE–AI may provide a practical pathway toward scalable, transparent, and verifiable clinical AI systems capable of supporting patient safety and quality improvement workflows in real-world healthcare environments.
