# Supplementary material for: A medically grounded LLM agent–based tool to detect patient safety events in medical records
Source: PLOS Digit Health. 2026 Sep 17;5(9):e0001174. doi: 10.1371/journal.pdig.0001174 (PMC13585220; doi:10.1371/journal.pdig.0001174)
Supplement: Supplementary file 1 [file pdig.0001174.s001.pdf]

Response to Reviewers

PLOS Digital Health | PDIG-D-25-01082

We thank the reviewers for their constructive feedback; noting the merit of our approach and its potential real-world application.

All manuscript line references in this response refer to the tracked-changes version of the manuscript and are also highlighted in blue.

Reviewer 1 - Major Concerns / Comments

We would like to thank the reviewer for making the time to write such thoughtful and useful comments. We have made substantive changes in response to these comments which we believe makes a much clearer and updated paper.

1. Statistical comparison methods

Reviewer comment: Methods & Results: This manuscript is missing details for statistical methods used for comparisons in the experiments. Results in Table 2 simply compute metrics but do not do statistical testing. Please revise to include an appropriate statistical comparison method in both methods and results.

Response: We have added a paired statistical comparison using McNemar's test to assess differences between our SAFE-AI framework and baselines on the same evaluation set and added a new Table –Table 3: "Paired McNemar comparison between SAFE-AI and baselines" to provide details of statistical methods. The results of this analysis have been included in the "Statistical analysis" subsection of the Results (lines 224-230).

2. LLM + ontology baseline description and comparison figure

Reviewer comment: Methods: Authors do not adequately describe how the LLM + Ontology baseline works. The lack of detail impedes understanding of where performance gain or failure may occur. Since ontology is extracted in the graph, is the LLM just provided the graph node relationships? It would greatly assist the reader's understanding if you provided an example figure which shows the same example being verified by all three experimental conditions LLM-only, LLM+Ontology, SAFE-AI.

Response: We have added a subsection in results titled "Ablated baselines", with paragraphs: "LLM-only baseline", "LLM with ontology baseline" and "SAFE-AI" (lines 160-184). We also added a new figure - Figure 4: "Overview of the compared baselines", where we describe in detail the differences of each baseline, highlighting how the LLM is prompted.

3. Correctness of guideline-to-code translation

Reviewer comment: Methods: Authors write "...the only source of hallucination stems from the initial information extraction...". While this is plausible, the author's method to then evaluate for ACEs involves addition of a computational graph and code execution. These introduce additional complexity and additional error surfaces which I believe to be under-characterized. For example, GPT-4o is asked to translate guidelines into code, but how do we know whether GPT-4o generated the correct validation code? Code compilers and interpreters can only guarantee logical correctness to execute, but not whether the correct logic is expressed. What if GPT-4o hallucinates or drifts from the original guideline and produces code that encodes logic that is different from what the guidelines state? Did authors verify that the code is correct? It would be nice to report % accuracy of how frequently GPT-4o is faithful/unfaithful to the original guidelines.

Response: We agree that, in principle, LLM-generated code could introduce errors if it does not reflect the underlying clinical guidelines.

In our framework, however, code generation is a one-time development step, and it is not part of the inference pipeline. GPT is used only to assist with translating clinical guidelines into Python functions offline. The generated code is then manually reviewed and verified by domain experts, fixed into a file, and reused across

all experiments. No code is generated at inference time. Thus, the deployed SAFE-AI system executes fully deterministic, where the only source of uncertainty at inference comes from the information extraction step.

We have clarified this distinction in the revised manuscript to avoid ambiguity in the Methods subsection “Ontologies as computational graphs” (lines120-129).

4. Error attribution across transformation stages

Reviewer comment: Methods: SAFE-AI introduces three steps for guideline transformation and enforcement--(1) creation of a formal ontology, (2) creation of a computational graph using the ontology, and (3) creation of python code from the computational graph. Each of these transformations may introduce some form of error. The ideal study would characterize the error rates (or lack thereof) across each stage of this process. Otherwise readers have no information about potential error attribution and which parts of the framework are strengths/weaknesses. Is there a way to examine these in greater detail?

Response: We thank the reviewer for this important suggestion. In the revised manuscript, we have expanded the analysis of our SAFE-AI behavior in two ways: First, we have added Table 4: “Taxonomy of failure modes in ASE detection”, where we report type, frequency, and interpretation of the observed failure modes of our approach. Second, we included a qualitative comparison of errors across baselines in the Results subsection “Epinephrine ASE detection” (Lines 186-222).

5. Frequency and recency bias discussion

Reviewer comment: Methods: There is much detail regarding LLM frequency bias and recency bias which feels tangential/unnecessary because there is no further experiment conducted to quantify how SAFE-AI mitigates these issues. Either provide an experiment/analysis to prove your point that SAFE-AI improves upon these issues or consider minimizing/removal of these topics as it has minimal relevance to the methods of your experimental results & findings. These points also feel misplaced as they are background/motivating factors for your proposed system rather than methods.

Response: We appreciate this insight. We have shortened the detail on LLM biases in one sentence and moved parts that seemed more like discussion than results to that section. In addition, we have simplified the Methods subsection “LLMs as zero-shot information extractors” to focus on the practical motivation for using an LLM as information extractors rather than reasoners. (Lines 80-118)

6. Backward chaining and subgraph selection

Reviewer comment: Methods: When determining relevant subgraph of nodes, authors describe that SAFE-AI uses a backwards chaining method. However, no details are provided here which leaves the reader unsure how much of the subgraph is actually selected. Are there any hyperparameters to this process? If so, was there any hyperparameter optimization, preliminary sensitivity analyses, or were they selected for specific reasons/intuitions?

Response: We agree with the reviewer, and we have added a more detailed description of our framework in Methods subsection “Ontologies as computational graphs” (paragraph two, lines 122-129; 131-134).

7. Clinician annotation disagreements

Reviewer comment: Results: In Abstract, Introduction, and Discussion, the authors allude to the fact that annotators had disagreements, but do not describe in detail. The creation of the annotated dataset, the interrater agreement, and the pattern of disagreements seen during the annotation process is a result of your study and should be reported in the Results section.

Response: We have added the Results subsection “Clinician annotation and inter-rater agreement” (lines 142-158) to describe where and why disagreements occurred. Additionally, we report clinician-clinician confusion matrices and percent agreement for each ASE category (Figure 3: Experts percent agreement for epinephrine ASEs).

8. Empty-field and incorrect-value extraction errors

Reviewer comment: Results: Authors identify Empty Field Errors & Incorrect Value Errors for LLM extraction phase. However, they do not analyze how this affects the final result. Are there patterns of failure? Are there

any ways SAFE-AI can "recover" from partial empty values? Is Overdose vs. Delay outcomes more frequently affected?

Response: Please refer to point 4.

9. Confidence intervals or uncertainty for Table 2

Reviewer comment: Results - Table 2: Trend supports author's conclusions that SAFE-AI performs better than baselines, but there is no estimation of uncertainty on the metrics in Table 2. Please report 95% confidence intervals or similar. I am concerned that due to the small annotated sample size, confidence intervals may be wide and the findings may not be statistically significant. If confidence intervals overlap, either increase your sample size or report that the findings are not statistically significant but there may be a trend toward significance.

Response: Although LLMs are stochastic in principle, all experiments were conducted under a fixed evaluation configuration (i.e., fixed dataset, identical prompts, and temperature set to a zero value). Empirically, we observed no variation in predictions across repeated runs. In this setting, classical confidence intervals are not informative. Instead, we report statistical significance using McNemar's test, which evaluates differences in correct predictions on the same set of clinical cases. Please refer to point 1.

10. LLM + ontology delay performance

Reviewer comment: Results - Table 2: Delay has 0% for precision and recall for LLM + Ontology, which is puzzling since LLM-only baseline achieves 75% precision and 80% recall. Please explain why adding the ontology would dramatically harm performance significantly. Without any explanation, this looks like an error and makes the reader question the rigor & veracity of all other findings.

Response: We thank the reviewer for pointing out this. During evaluation, we identified and corrected an inconsistency in the temporal parsing component affecting delay computation. In addition, the original experiments were conducted using a GPT-4-family model that has since been deprecated and replaced by the currently available GPT-5.4 mini model. As the GPT-4 family is no longer accessible under the same API conditions, we re-ran all experiments using the current stable model version to guarantee reproducibility and consistency with the present API environment.

We observe some degree of variability across model families, particularly borderline cases of delay detection, which speaks to the sensibility of LLM reasoning to model versions. It can be observed that adding the ontology can reduce performance in such cases due to its structural rigidity. Specifically, errors in entity extraction or temporal alignment may propagate through the ontology, leading to misclassification even when partial information is correct. In contrast, the LLM-only baseline, while less constrained, can sometimes recover from incomplete representations via direct contextual inference. Despite this, our SAFE-AI remains more stable across model changes, as it separates probabilistic information extraction from deterministic rule-based execution one entities are grounded. We have updated text in accordance with this change in GPT.

For more details on this, please refer to the added Results subsection "Ontological prompting improves consistency but introduces conservative behavior" (lines 202-211).

11. Qualitative examples of baseline failures

Reviewer comment: Results: Currently the discussion of LLM-only predictions & LLM+Ontology findings simply report verbatim what is already stated in Table 2 and does not provide additional value to the reader. Instead, please review specific examples which contribute to lower performance in these baselines which SAFE-AI overcomes and highlight patterns of behavior in which SAFE-AI improves upon the baselines. This provides readers with insight and intuition on why SAFE-AI performs better or how LLM-only and LLM+Ontology fails.

Response: We agree with the reviewer that simply restating quantitative results does not provide sufficient insight into our model behavior. We have added the Results subsections "Epinephrine ASE detection" (lines 186-222), and "Error analysis" (lines 232-242) to include a qualitative error analysis.

Reviewer 1 - Minor Concerns / Comments

1. Introduction should distinguish SAFE-AI from generic RAG

Reviewer comment: Introduction: Authors describe SAFE-AI at a high-level but use very generic language, describing it as a RAG-based system using ontologies. There are many ways to design RAG-based systems, many of which do not use a graph let alone a computational graph that involves code execution. I recommend authors revise the introduction to highlight the unique salient aspects of SAFE-AI (e.g. computational graph creation, transformation of guidelines to computer code, etc.) so readers understand how the proposed approach is differentiated from vanilla RAG or other information-retrieval based approaches.

Response: We have clarified the distinction between the proposed SAFE-AI and retrieval-based approaches such as RAG. In particular, we highlight that RAG is a generation paradigm where the LLM performs reasoning, in contrast, SAFE-AI separates extraction from the decision-making process by using deterministic, rule-based computation over structured variables. (Lines 40-41)

2. Dataset size and narrow task limitations

Reviewer comment: Discussion: Limitations should include the fact that SAFE-AI is only tested on a small dataset of 300 cases and only two tasks (epinephrine timing and dose). While these are important tasks, it's unclear how the system would perform on detecting other ASE which are not medication-related in nature such as pressure ulcers, defective/contaminated devices, wrong-site surgery, etc.

3. Dependence on clinical guidelines

Reviewer comment: Discussion: This uses a RAG-driven approach which relies on guidelines. This assumes that guidelines exist for every domain for ASE. Please comment/discuss on whether this is the case; if not, please raise this as a limitation of your method.

4. Pediatric EMS scope and generalizability

Reviewer comment: Discussion: Why limit it to only pediatric EMS events? While this is an important use case, the limited diversity of events in the dataset greatly impedes an argument for the generalizability of the SAFE-AI method.

Overall Response: We have revised the Discussion section to include the limitations of our proposed methods in all these aspects (lines 294-305).

Reviewer 2

1. Wording that sounds LLM-generated

Reviewer comment: There are so many phrases in the manuscript, such as LLM (ChatGPT) generated, such as professionals' abilities (line 10), system's performance (line 4), and so on. Could the authors explain and revise them?

Response: We thank the reviewer for this observation. We would like to clarify that the manuscript was not generated using ChatGPT or any other large language model. We understand that certain stylistic patterns may appear unnatural. To address this concern, we have carefully revised the manuscript to improve clarity and adopt more conventional academic phrasing. (Abstract line 10; lines 4, 10-12, 20, 25, 28-29, 42, 44, 263-264, 323-324).

2. SAFE-AI versus clinician reviewer disagreements

Reviewer comment: Could authors explain the disagreements between the SAFE-AI predictions and the clinician reviewers, or were errors made by the model? How different are the views of clinician reviewers?

Response: We have revised and extended the Results section of the manuscript to characterize SAFE-AI predictions and clinician inter-rater agreement by including the subsections “Clinician annotation and inter-rater agreement” (lines 145-151), “Error analysis” (lines 233-242), Table 4 “Taxonomy of failure modes in ASE detection”, and the overall Discussion section (lines 248-252).

3. Agentic AI limitations and future improvement

Reviewer comment: SAFE-AI has limitations with Agentic AI. Could authors explain how to improve the SAFE-AI using the Agentic Ai approach?

Response: We thank the reviewer for this insightful suggestion. The main objective of our proposed SAFE-AI framework is to improve the current process for identifying adverse safety events (ASEs), which requires manual expert review. In this sense, our work represents a step toward both scalable and interpretable detection by combining LLM’s capability to extract information regardless of format and deterministic reasoning based on guidelines.

We agree that agentic AI approaches could bring additional advantages in future extensions of the framework. For example, by including a multi-step verification of the extracted entities and the generation of ontologies for other clinical domains. At the same time, we note that modern agentic frameworks continue to rapidly evolve, and this work was practically completed before their widespread adoption. As the field continues to mature and stabilize, we believe agentic approaches may become more suitable for clinical review tasks such as the identification of ASEs.

The proposed work intentionally prioritized deterministic and interpretable decision-making over autonomous reasoning, which are critical in clinical settings. However, as future work we plan to incorporate agentic capabilities while keeping clinically grounded processes.

4. Code script and video demo

Reviewer comment: To increase the transparency and reliability, could authors share the Python code script and video demo on how SAFE-AI works?

Response: We have made our code repository publicly available and added the corresponding link (https://github.com/dietrujillo/medically_grounded_llm) to the Declarations subsection “Access to Data” (lines 325-326).
