# Supplementary material for: A medically grounded LLM agent–based tool to detect patient safety events in medical records
Source: PLOS Digit Health. 2026 Sep 17;5(9):e0001174. doi: 10.1371/journal.pdig.0001174 (PMC13585220; doi:10.1371/journal.pdig.0001174)
Supplement: Peer review history

# Peer review: Decision Letter 0

**DOI:** 10.1371/journal.pdig.0001174.r001

**Authors:** Xiaoli Liu, Zhenwei Shi

5 Mar 2026

Response to Reviewers Revised Manuscript with Track Changes Manuscript **Journal Requirements:**

1. Please provide a detailed online Financial Disclosure statement. This is published with the article. It must therefore be completed in full sentences and contain the exact wording you wish to be published.

a) State the initials, alongside each funding source, of each author to receive each grant. For example: “This work was supported by the National Institutes of Health (####### to AM; ###### to CJ) and the National Science Foundation (###### to AM).”

b) State what role the funders took in the study. If the funders had no role in your study, please state: “The funders had no role in study design, data collection and analysis, decision to publish, or preparation of the manuscript.”

For more information, please go to our submission guidelines:

https://journals.plos.org/digitalhealth/s/submission-guidelines#loc-financial-disclosure-statement

2. Please ensure that the funders and grant numbers match between the Financial Disclosure field and the Funding Information tab in your submission form. Note that the funders must be provided in the same order in both places as well.

3. Please update your online Competing Interests statement. If you have no competing interests to declare, please state: "The authors have declared that no competing interests exist."

4. For studies involving third-party data, we encourage authors to share any data specific to their analyses that they can legally distribute. PLOS recognizes, however, that authors may be using third-party data they do not have the rights to share. When third-party data cannot be publicly shared, authors must provide all information necessary for interested researchers to apply to gain access to the data. (https://journals.plos.org/digitalhealth/s/data-availability#loc-acceptable-data-access-restrictions)

For any third-party data that the authors cannot legally distribute, they should include the following information in their Data Availability Statement upon submission:

a) A description of the data set and the third-party source

b) If applicable, verification of permission to use the data set

c) Confirmation of whether the authors received any special privileges in accessing the data that other researchers would not have

d) All necessary contact information others would need to apply to gain access to the data

5. Please upload your main article file as a .doc, .docx or .rtf file.

6. Please provide separate main figure files in .tif or .eps format only and ensure that all files are under our size limit of 10MB.

For more information about how to convert your figure files please see our guidelines: https://journals.plos.org/digitalhealth/s/figures

7. If the reviewer comments include a recommendation to cite specific previously published works, please review and evaluate these publications to determine whether they are relevant and should be cited. There is no requirement to cite these works unless the editor has indicated otherwise.

**Additional Editor Comments (if provided):**

Thank you for your patience. The reviewers' assessments are consistent, so we are pleased to send you the comments. We look forward to receiving your revised manuscript.

Best wishes,

**Reviewers' Comments:**

**Comments to the Author**

1. Does this manuscript meet PLOS Digital Health’s publication criteria?

Reviewer #1: Partly

Reviewer #2: Yes

**********

2. Has the statistical analysis been performed appropriately and rigorously?

Reviewer #1: No

Reviewer #2: N/A

**********

3. Have the authors made all data underlying the findings in their manuscript fully available (please refer to the Data Availability Statement at the start of the manuscript PDF file)?

The PLOS Data policy

Reviewer #1: Yes

Reviewer #2: No

**********

4. Is the manuscript presented in an intelligible fashion and written in standard English?

Reviewer #1: Yes

Reviewer #2: Yes

**********

Reviewer #1: Overview

Authors present SAFE-AI, an agentic LLM system that claims to reduce hallucinations by leveraging clinician-expert guidelines, transforming these guidelines into a computational graph using custom ontologies, and subsequent transformation of subgraphs selected at runtime into python code functions which are executed to detect adverse safety events. The authors evaluate the system on a pediatric epinephrine ASE detection task in 300 EMS clinical charts for which the comparison ground truth was established by two physicians independently annotating for ASE. While the approach is interesting and may have merit, there are significant gaps in the SAFE-AI system description, characterizations of potential failure modes introduced by system design, and identifying reasons for errors/failures from baseline methods vs. SAFE-AI. Addition of experiments and analysis that quantify these facets of SAFE-AI would dramatically improve the understanding of system performance and its potential real-world application in ASE detection in retrospective chart review. My comments and concerns are listed below.

If my comments/concerns are unclear, feel free to reach out to me directly: Philip Chung, MD, MS (chungp@stanford.edu).

Major Concerns/Comments

1. Methods & Results: This manuscript is missing details for statistical methods used for comparisons in the experiments. Results in Table 2 simply compute metrics but do not do statistical testing. Please revise to include an appropriate statistical comparison method in both methods and results.

2. Methods: Authors do not adequately describe how the LLM + Ontology baseline works. The lack of detail impedes understanding of where performance gain or failure may occur. Since ontology is extracted in the graph, is the LLM just provided the graph node relationships? It would greatly assist the reader’s understanding if you provided an example figure which shows the same example being verified by all three experimental conditions LLM-only, LLM+Ontology, SAFE-AI.

3. Methods: Authors write “...the only source of hallucination stems from the initial information extraction…”. While this is plausible, the author’s method to then evaluate for ACEs involves addition of a computational graph and code execution. These introduce additional complexity and additional error surfaces which I believe to be under-characterized. For example, GPT-4o is asked to translate guidelines into code, but how do we know whether GPT-4o generated the correct validation code? Code compilers and interpreters can only guarantee logical correctness to execute, but not whether the correct logic is expressed. What if GPT-4o hallucinates or drifts from the original guideline and produces code that encodes logic that is different from what the guidelines state? Did authors verify that the code is correct? It would be nice to report % accuracy of how frequently GPT-4o is faithful/unfaithful to the original guidelines.

4. Methods: SAFE-AI introduces three steps for guideline transformation and enforcement–(1) creation of a formal ontology, (2) creation of a computational graph using the ontology, and (3) creation of python code from the computational graph. Each of these transformations may introduce some form of error. The ideal study would characterize the error rates (or lack thereof) across each stage of this process. Otherwise readers have no information about potential error attribution and which parts of the framework are strengths/weaknesses. Is there a way to examine these in greater detail?

5. Methods: There is much detail regarding LLM frequency bias and recency bias which feels tangential/unnecessary because there is no further experiment conducted to quantify how SAFE-AI mitigates these issues. Either provide an experiment/analysis to prove your point that SAFE-AI improves upon these issues or consider minimizing/removal of these topics as it has minimal relevance to the methods of your experimental results & findings. These points also feel misplaced as they are background/motivating factors for your proposed system rather than methods.

6. Methods: When determining relevant subgraph of nodes, authors describe that SAFE-AI uses a backwards chaining method. However, no details are provided here which leaves the reader unsure how much of the subgraph is actually selected. Are there any hyperparameters to this process? If so, was there any hyperparameter optimization, preliminary sensitivity analyses, or were they selected for specific reasons/intuitions?

7. Results: In Abstract, Introduction, and Discussion, the authors allude to the fact that annotators had disagreements, but do not describe in detail. The creation of the annotated dataset, the interrater agreement, and the pattern of disagreements seen during the annotation process is a result of your study and should be reported in the Results section.

8. Results: Authors identify Empty Field Errors & Incorrect Value Errors for LLM extraction phase. However, they do not analyze how this affects the final result. Are there patterns of failure? Are there any ways SAFE-AI can “recover” from partial empty values? Is Overdose vs. Delay outcomes more frequently affected?

9. Results - Table 2: Trend supports author’s conclusions that SAFE-AI performs better than baselines, but there is no estimation of uncertainty on the metrics in Table 2. Please report 95% confidence intervals or similar. I am concerned that due to the small annotated sample size, confidence intervals may be wide and the findings may not be statistically significant. If confidence intervals overlap, either increase your sample size or report that the findings are not statistically significant but there may be a trend toward significance.

10. Results - Table 2: Delay has 0% for precision and recall for LLM + Ontology, which is puzzling since LLM-only baseline achieves 75% precision and 80% recall. Please explain why adding the ontology would dramatically harm performance significantly. Without any explanation, this looks like an error and makes the reader question the rigor & veracity of all other findings.

11. Results: Currently the discussion of LLM-only predictions & LLM+Ontology findings simply report verbatim what is already stated in Table 2 and does not provide additional value to the reader. Instead, please review specific examples which contribute to lower performance in these baselines which SAFE-AI overcomes and highlight patterns of behavior in which SAFE-AI improves upon the baselines. This provides readers with insight and intuition on why SAFE-AI performs better or how LLM-only and LLM+Ontology fails.

Minor Concerns/Comments

1. Introduction: Authors describe SAFE-AI at a high-level but use very generic language, describing it as a RAG-based system using ontologies. There are many ways to design RAG-based systems, many of which do not use a graph let alone a computational graph that involves code execution. I recommend authors revise the introduction to highlight the unique salient aspects of SAFE-AI (e.g. computational graph creation, transformation of guidelines to computer code, etc.) so readers understand how the proposed approach is differentiated from vanilla RAG or other information-retrieval based approaches.

2. Discussion: Limitations should include the fact that SAFE-AI is only tested on a small dataset of 300 cases and only two tasks (epinephrine timing and dose). While these are important tasks, it’s unclear how the system would perform on detecting other ASE which are not medication-related in nature such as pressure ulcers, defective/contaminated devices, wrong-site surgery, etc.

3. Discussion: This uses a RAG-driven approach which relies on guidelines. This assumes that guidelines exist for every domain for ASE. Please comment/discuss on whether this is the case; if not, please raise this as a limitation of your method.

4. Discussion: Why limit it to only pediatric EMS events? While this is an important use case, the limited diversity of events in the dataset greatly impedes an argument for the generalizability of the SAFE-AI method.

Reviewer #2: Comments for the Manuscript

Thank you for the opportunity to review the manuscript, “A medically-grounded LLM agent–based tool to detect patient safety events in medical records.”

The researchers have developed SAFE–AI for clinical decision making on epinephrine adverse safety events (ASEs). The criteria for severe epinephrine adverse safety events (ASEs) are presented clearly. The results of SAFE–AI demonstrated high accuracy compared with the LLM-Only Baseline and the LLM + Ontology. Thus, SAFE–AI illustrates potential clinical AI applications.

However, there are some comments to the authors:

-There are so many phrases in the manuscript, such as LLM (ChatGPT) generated, such as professionals’ abilities (line 10), system’s performance (line 4), and so on

Could the authors explain and revise them?

-Could authors explain the disagreements between the SAFE-AI predictions and the clinician reviewers, or were errors made by the model? How different are the views of clinician reviewers?

-SAFE-AI has limitations with Agentic AI. Could authors explain how to improve the SAFE-AI using the Agentic Ai approach?

- To increase the transparency and reliability, could authors share the Python code script and video demo on how SAFE–AI works?

Accepted with the revision

**********

what does this mean?). If published, this will include your full peer review and any attached files.

**Do you want your identity to be public for this peer review?**  If you choose “no”, your identity will remain anonymous but your review may still be made public.

For information about this choice, including consent withdrawal, please see our Privacy Policy

Reviewer #1: **Yes:**  Philip Chung

Reviewer #2: No

**********

**Figure resubmission:**

**Reproducibility:**

To enhance the reproducibility of your results, we recommend that authors of applicable studies deposit laboratory protocols in protocols.io, where a protocol can be assigned its own identifier (DOI) such that it can be cited independently in the future. Additionally, PLOS ONE offers an option to publish peer-reviewed clinical study protocols. Read more information on sharing protocols at https://plos.org/protocols?utm_medium=editorial-email&utm_source=authorletters&utm_campaign=protocols

# Author comment: Author response to Decision Letter 1

**DOI:** 10.1371/journal.pdig.0001174.r002

1 Jul 2026

Attachment Submitted filename: PDIG-D-25-01082_Response_to_Reviewers.pdf

# Peer review: Decision Letter 1

**DOI:** 10.1371/journal.pdig.0001174.r003

**Authors:** Xiaoli Liu, Zhenwei Shi

23 Jul 2026

A medically-grounded LLM agent–based tool to detect patient safety events in medical records

PDIG-D-25-01082R1

Dear Dr Guise,

We are pleased to inform you that your manuscript 'A medically-grounded LLM agent–based tool to detect patient safety events in medical records' has been provisionally accepted for publication in PLOS Digital Health.

Before your manuscript can be formally accepted you will need to complete some formatting changes, which you will receive in a follow-up email from a member of our team.

Please note that your manuscript will not be scheduled for publication until you have made the required changes, so a swift response is appreciated.

IMPORTANT: The editorial review process is now complete. PLOS will only permit corrections to spelling, formatting or significant scientific errors from this point onwards. Requests for major changes, or any which affect the scientific understanding of your work, will cause delays to the publication date of your manuscript.

If your institution or institutions have a press office, please notify them about your upcoming paper to help maximize its impact. If they'll be preparing press materials, please inform our press team as soon as possible -- no later than 48 hours after receiving the formal acceptance. Your manuscript will remain under strict press embargo until 2 pm Eastern Time on the date of publication. For more information, please contact digitalhealth@plos.org.

Thank you again for supporting Open Access publishing; we are looking forward to publishing your work in PLOS Digital Health.

Best regards,

Xiaoli Liu, PhD

Academic Editor

PLOS Digital Health

***********************************************************

**Additional Editor Comments (if provided):**

Dear Dr. Guise,

Great work! We look forward to seeing your article published and read by the audience soon.

Best wishes,

Dr. Xiaoli Liu

**Reviewer Comments (if any, and for reference):**

Reviewer's Responses to Questions

**Comments to the Author**

Reviewer #1: All comments have been addressed

Reviewer #2: All comments have been addressed

**********

publication criteria?

Reviewer #1: Yes

Reviewer #2: Yes

**********

3. Has the statistical analysis been performed appropriately and rigorously?

Reviewer #1: Yes

Reviewer #2: Yes

**********

4. Have the authors made all data underlying the findings in their manuscript fully available (please refer to the Data Availability Statement at the start of the manuscript PDF file)?

The PLOS Data policy

Reviewer #1: Yes

Reviewer #2: Yes

**********

5. Is the manuscript presented in an intelligible fashion and written in standard English?

Reviewer #1: Yes

Reviewer #2: Yes

**********

Reviewer #1: Thank you for addressing my concerns. This version of the manuscript is substantially clearer and improved.

Reviewer #2: Authors addressed all my comments. I recommend the manuscript for publication.

**********

what does this mean?). If published, this will include your full peer review and any attached files.

**Do you want your identity to be public for this peer review?**  If you choose “no”, your identity will remain anonymous but your review may still be made public.

For information about this choice, including consent withdrawal, please see our Privacy Policy

Reviewer #1: **Yes:**  Philip Chung

Reviewer #2: No

**********
